# Supplementary material for: Melatonin Inhibits VEGF-Induced Endothelial Progenitor Cell Angiogenesis in Neovascular Age-Related Macular Degeneration
Source: Cells. 2023 Mar 3;12(5):799. doi: 10.3390/cells12050799 (PMC10000467; doi:10.3390/cells12050799)
Supplement: Supplementary file 1 [file cells-12-00799-s001.zip › cells-2203982-supplementary.pdf]

Supplementary Methods

MTT assay

EPCs were stimulated with varying concentrations of melatonin (0.1, 0.3, or 1 mM) for 24 h or 48 h, then washed with PBS and treated with 3-(4,5-dimethylthiazol-2-yl)-2,5-diphenyltetrazolium bromide (MTT; 0.5 mg/ml) for 4 h. After confirming the formation of formazan crystals, EPCs were dissolved in dimethylsulfoxide (DMSO). Absorbance at 570 nm was determined by a microplate reader (Bio-Tek, Winooski, VT, USA).[1]

Western blot analysis

Cell lysates were collected using RIPA buffer. Proteins were resolved using SDS-PAGE then transferred to Immobilon-P 0.45 μm polyvinylidene difluoride membranes. The membranes were blocked with 5% non-fat milk for 1 h, then treated with primary antibodies (1:1000). After undergoing 3 washes with TBST, the membranes were treated with secondary antibodies (1:3000) then visualized using the ImageQuant™ LAS 4000 biomolecular imager (GE Healthcare Life Sciences, USA).[2]

Luciferase reporter assay

EPCs were transfected with the NF-κB or AP-1 luciferase plasmids (Stratagene, La Jolla, CA, USA) using Lipofectamine 2000, then treated with VEGF and melatonin. Cell lysates were examined using the reporter lysis buffer and luciferase assay substrate. Luciferase activity was determined using our established protocol.[3]

Supplementary Results

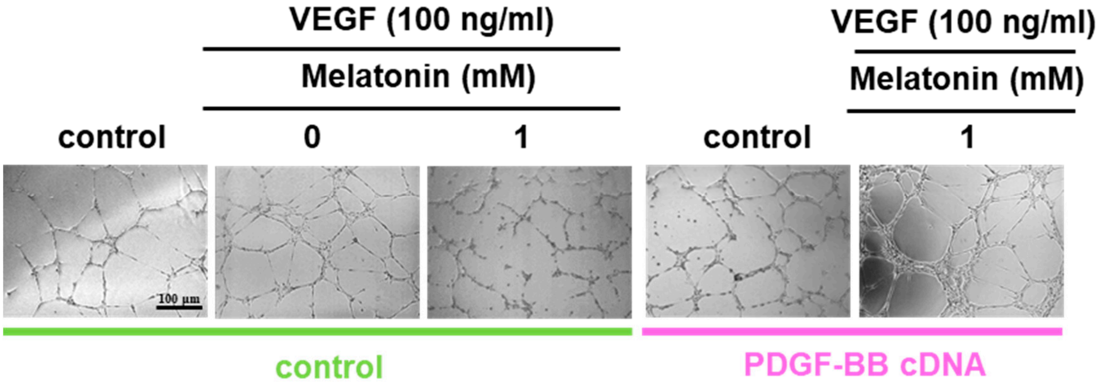

Supplementary Figure S1. Melatonin suppresses tube formation by inhibiting PDGF-BB expression in EPCs. EPCs were transfected with PDGF-BB cDNA overnight, then left untreated or were treated with VEGF (100 ng/ml) and melatonin (1 mM) for 24 h, before examination with tube formation assays (n=3).

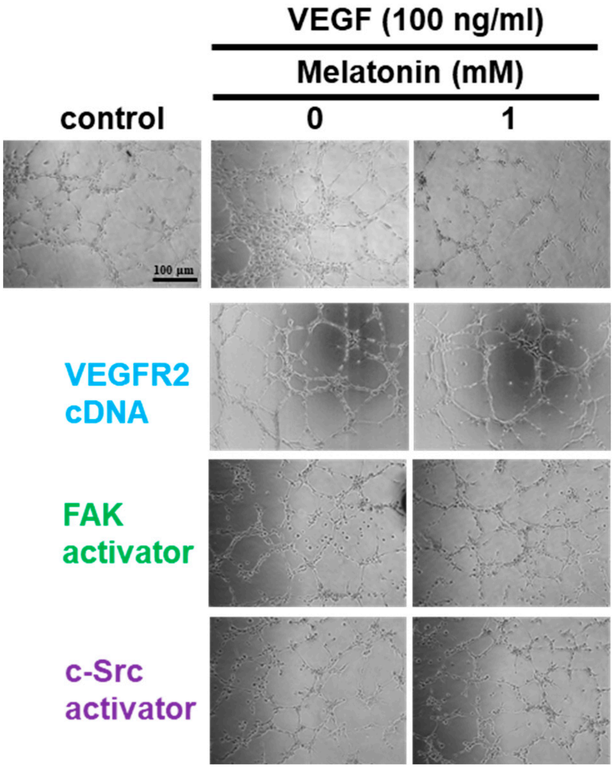

Supplementary Figure S2. Melatonin suppresses tube formation by inhibiting VEGFR2, c-Src, and FAK expression in EPCs. EPCs were transfected with VEGFR2 cDNA or treated with FAK or c-Src activators overnight, then left untreated or were treated with VEGF (100 ng/ml) alone and in combination with melatonin (1 mM) for 24 h, before examination with tube formation assays (n=4).

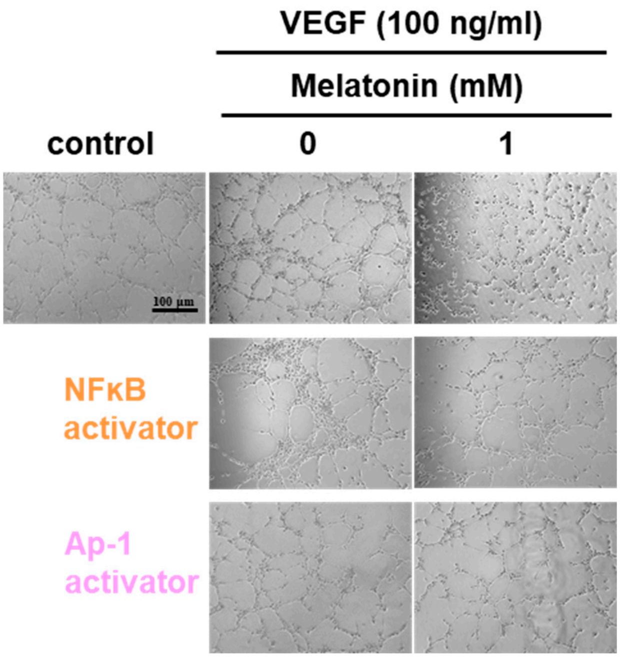

Supplementary Figure S3. Melatonin suppresses tube formation by inhibiting NF-κB and AP-1 expression in EPCs. EPCs were treated with NF-κB or AP-1 activators overnight, then left untreated or were treated with VEGF (100 ng/ml) alone and in combination with melatonin (1 mM) for 24 h, before examination with tube formation assays (n=4).

References

1. Liu S-C, Tsai C-H, Wu T-Y, et al. Soya-cerebroside reduces IL-1β-induced MMP-1 production in chondrocytes and inhibits cartilage degradation: implications for the treatment of osteoarthritis. *Food and Agricultural Immunology.* 2019;30(1):620-632.
2. Achudhan D, Li-Yun Chang S, Liu SC, et al. Antcin K inhibits VCAM-1-dependent monocyte adhesion in human rheumatoid arthritis synovial fibroblasts. *Food Nutr Res.* 2022;66.
3. Wu KM, Hsu YM, Ying MC, et al. High-density lipoprotein ameliorates palmitic acid-induced lipotoxicity and oxidative dysfunction in H9c2 cardiomyoblast cells via ROS suppression. *Nutr Metab (Lond).* 2019;16:36.
